# Elevated Soluble HLA‐G Levels Associate With Dengue Severity in Vietnamese Patients

**DOI:** 10.1002/jmv.70594

**Published:** 2025-09-05

**Authors:** Do Duc Anh, Nguyen Trong The, Le Huu Song, Anja Mueller, Thirumalaisamy P. Velavan, Barbara Seliger

**Affiliations:** ^1^ Institute of Tropical Medicine University of Tübingen Tübingen Germany; ^2^ Vietnamese ‐ German Centre for Medical Research (VG‐CARE) Hanoi Vietnam; ^3^ 108 Military Central Hospital Hanoi Vietnam; ^4^ Medical Faculty Martin Luther University Halle‐Wittenberg Halle (Saale) Germany; ^5^ Faculty of Medicine Duy Tan University Da Nang Vietnam; ^6^ Institute of Translational Immunology and Centre of Translational Medicine Brandenburg; ^7^ Medical School “Theodor Fontane” Brandenburg an der Havel Germany; ^8^ Faculty of Health Sciences Brandenburg, Brandenburg Medical School “Theodor Fontane”, Institute of Translational Immunology Brandenburg Germany; ^9^ Fraunhofer Institute for Cell Therapy and Immunology Leipzig Germany

**Keywords:** clinical severity, dengue, sHLA‐G, Vietnam

## Abstract

The pathogenesis of dengue remains complex and incompletely understood. One proposed mechanism involves the virus evading host immune responses through the upregulation and/or secretion of immune‐inhibitory molecules. This study investigates the association between plasma levels of soluble human leukocyte antigen G (sHLA‐G), a known immunoregulatory molecule, and dengue severity in hospitalized patients. A total of 238 dengue patients and 118 healthy controls were enrolled. Dengue infection was confirmed by real‐time RT‐PCR, and patients were clinically categorized as having dengue fever without warning signs (DF), dengue with warning signs (DWS), or severe dengue (SD), according to WHO guidelines. Laboratory parameters were assessed upon hospital admission, and plasma sHLA‐G levels were measured using ELISA. sHLA‐G levels were significantly elevated in dengue patients compared to healthy controls (median [range]: 42.7 [7.10–1300] U/mL vs. 11.1 [4.7–620] U/mL; *p*  <  0.001). After adjusting for age, sex and disease severity, a significant association was observed between sHLA‐G levels and days of illness (*β* = 0.1, *p* = 0.03). Patients requiring close medical monitoring (DWS/SD) showed higher sHLA‐G levels (51.0 [7.17–525] U/mL) than those having dengue fever without warning signs (38.0 [7.10–1300] U/mL); *p* = 0.011. While ALT and AST were positively correlated with sHLA‐G levels in all patients, total lymphocyte counts were inversely correlated with sHLA‐G in severe cases (*r* = −0.78, *p* = 0.002). Elevated sHLA‐G levels are associated with dengue severity and may serve as a useful marker for identifying high‐risk cases and for guiding clinical monitoring.

**Clinical trial registration:** Not applicable.

## 1 Introduction

The dengue virus (DENV) is responsible for massive outbreaks of viral haemorrhagic fever worldwide, particularly in tropical and subtropical regions [1]. Vietnam experiences a high annual incidence of dengue with a significant impact on the health and economic system [2]. Clinically, the disease can range from nonspecific acute febrile illness to haemorrhagic manifestations with altered haemostasis, possibly leading to shock or multiorgan failure [3]. The pathogenesis of dengue is complex and has not yet been elucidated in detail, as it depends on both viral and host factors, with the host immune system playing a decisive role.

After infection, dendritic cells (DCs) presenting the DENV antigen recruit monocytes and macrophages to the site of infection. Instead of effectively eliminating the virus, these recruited immune cells ‐ important components of the innate immune response ‐ become primary targets for DENV [4]. This process facilitates viral replication and dissemination to other host immune cells in the lymph nodes, bone marrow, spleen and liver. The molecular and immunological mechanisms by which DENV subverts the host's immune response to viral antigens, remains unclear.

One possible explanation is that DENV preferentially infects DCs, monocytes and macrophages thereby impairing the development of an efficient early antiviral response. During viral replication, DENV secretes nonstructural proteins (NS), specifically NS1, NS2B and NS4, which disrupt the signalling pathway of type I interferon (IFN), a critical component of the innate immune defence [5]. This disruption delays the host's antiviral response and allows the virus to persist and propagate. In addition, secondary DENV infections can lead to antibody‐dependent enhancement, in which non‐neutralising antibodies facilitate viral replication and thus increase the disease burden for the host [5].

Furthermore, the production of immune checkpoints (ICP) during DENV infection can impair the host's immune response [6, 7]. These molecules trigger T cells dysfunction and inhibit immune cells activity and are associated with impaired immune defence in various diseases, including cancer and infectious diseases [7, 8]. One of the potential immune inhibitors is the HLA‐G ligand (Human Leukocyte Antigens G), which is a ligand for receptors in B cells, T cells, monocytes, DCs and subsets of natural killer cells (NKs) [9]. HLA‐G molecules are involved in the inhibition of NK cells activity, the maturation of CD4 + T lymphocytes and DCs, the apoptosis of CD8+ cytotoxic T cells and the development of regulatory T cells (Tregs) [9].

The expression and regulation of HLA‐G is highly dynamic: four membrane‐bound forms (HLA‐G1 to G4) and three soluble, secreted forms (sHLA‐G5 to G7) generated by alternative splicing of the primary transcript [10]. The HLA‐G1 transmembrane isoform can produce a soluble form (sHLA‐G) by proteolytic shedding, which retains all the functions of the membrane counterpart, potentially expanding immunoregulatory activities on a systematic scale. Studies have reported an upregulation of HLA‐G antigens upon DENV infections suggesting a role of the ICPs in the pathogenesis of dengue [11]. Furthermore, it has also been observed that the level of sHLA‐G is modulated at different stages in arboviral infections [6].

While the role of HLA‐G in organ transplantation, pregnancy and cancer is well documented, its involvement in viral infections has not yet been sufficiently studied. This study examines sHLA‐G levels in dengue patients to assess their association with disease severity.

## 2 Methods

### 2.1 Ethical Approval Statement

All study participants provided signed informed consent before enrolment. The Institutional Review Board of the 108 Military Hospital and the University of Tübingen approved the study, titled “Host and Viral Factors Influencing Dengue Severity and Susceptibility” (Ethics Approval No. 274/2022B02). The study complies with the Nagoya Protocol and authorization for the use of genetic resources in Germany was obtained from the Vietnamese Ministry of Natural Resources and Environment (Reference No. 2995/QĐ‐BTNMT). All procedures followed GCP/GCLP guidelines.

### 2.2 Study Population

Samples were collected during two consecutive seasonal outbreaks in northern Vietnam, spanning September to November in 2021 and 2022. The study population consisted of 238 civilian patients suspected of having dengue who agreed to be enrolled in the study and were admitted to the 108 Military Central Hospital in Hanoi. The dengue diagnoses followed the World Health Organisation diagnostic's criteria [3] (https://apps.who.int/iris/handle/10665/44188), as adopted by the Vietnamese Ministry of Health. The inclusion criteria are patients presenting with fever and at least two clinical signs or symptoms suggestive of dengue (e.g. nausea/vomiting, rash, body aches and pains, positive tourniquet test) and/or a positive result from at least one indirect diagnostic method (serological rapid test), as recommended in the WHO 2009 guidelines [3]. Patients with bacterial or other viral infections, chronic diseases, or haematological disorders were excluded. A total of 118 Vietnamese healthy blood donors were recruited and considered as healthy controls in the study. Blood samples were collected at admission and plasma was separated and stored at −70°C until analysis.

### 2.3 PCR Confirmation of Dengue

Total viral RNA was isolated from 140 µL of patient plasma utilizing the QIAmp Viral RNA Mini Kit (Qiagen GmbH, Hilden, Germany) following the manufacturer's instructions. All samples (*n* = 238) underwent multiplex real‐time PCR analysis for dengue, Zika, and chikungunya viral RNA using the Fast‐Track Diagnostics Kit (Siemens Healthcare GmbH, Erlangen, Germany) on a LightCycler480‐II (Roche, Mannheim, Germany), following the manufacturer's guidelines. Testing was performed using internal controls and provided standards from the manufacturer. Confirmed dengue cases were identified by the presence of dengue viral RNA through real‐time RT‐PCR and absence of Zika and chikungunya viral RNA.

### 2.4 Dengue Severity Classification and Laboratory Assessment

In Vietnam, admitted patients were clinically classified into three severity levels according to WHO guidelines [3]: dengue without warning signs (DF, *n* = 103), dengue with warning signs (DWS, *n* = 122) and severe dengue (SD, *n* = 13). In this classification, warning signs include the presence of abdominal pain, persistent vomiting, mucosal bleeding, clinical fluid accumulation, lethargy/restlessness, liver enlargement and a rapid decrease in platelet count with rising haematocrit. Severe dengue is defined by severe plasma leakage leading to shock or respiratory distress, severe bleeding or severe organ impairment such as hepatitis, myocarditis or encephalopathy [3]. Patients who required close monitoring and hospitalization included both those with DWS and SD, as recommended by the WHO [3]. Accordingly, these groups were combined for the analysis of sHLA‐G levels to highlight clinically relevant differences and ensure statistical power. Clinical presentations were documented upon admission. Laboratory parameters assessed during admission include a complete hemogram: Erythrocyte (RBC), Haemoglobin (Hb), Haematocrit (HCT), Platelet (PLT), Leucocyte (WBC), Neutrophile (NEU), Lymphocyte (LYM), Monocyte (MONO), Eosinophile (EOS), Basophile (BASO), Large Unstained Cells (LUC), and liver enzymes: Aspartate Aminotransferase (AST) and Alanine Aminotransferase (ALT) levels.

### 2.5 Measurement of sHLA‐G Plasma Levels

Plasma levels of sHLA‐G (sHLA‐G1 and sHLA‐G5) were quantified in 238 patients and 118 healthy controls using the sHLA‐G enzyme‐linked immunosorbent assay (ELISA) kit (BioVendor–Laboratorní medicína a.s., Brno, Czech Republic), a sandwich enzyme immunoassay for the quantitative measurement of sHLA‐G, according to the manufacturer's instructions, whose detection limit is 0.38 ng/mL. Absorbance was measured using an ELISA reader (Infinite 200 PRO‐TECAN, Maennedorf, Switzerland) at 450 nm with the reference wavelength set to 630 nm. sHLA‐G concentrations were determined from a five‐point standard curve prepared from serial dilutions of calibrator (7.81, 15.63, 31.25, 62.5, and 125 U/mL) purchased by the kit as standard reagent. Results were expressed as Units/millilitre (U/mL).

### 2.6 Statistical Analysis

Data were analysed and visualized using the R software version 4.3.2 (http://www.r-project.org). Clinical and demographic data were presented as median values (with range) for quantitative variables and absolute numbers with percent for categorical variables. The normality of distribution in the quantitative variables was tested using the Shapiro‐Wilk test. Categorical data were compared using Chi‐square test, while continuous variables were compared using Kruskal‐Wallis or Wilcoxon test as appropriate. Dunn test was applied as post‐hoc test. Spearman correlation with Bonferroni adjustment was applied to assess the correlation between levels of sHLA‐G and other blood parameters. A *p* < 0.05 was considered statistically significant.

Multiple linear regression was performed to estimate the relationship between sHLA‐G levels and days of illness, adjusted for age, sex and clinical severity. In our study, days of illness are defined as the number of days from the onset of fever until admission. The sHLA‐G values were log‐transformed before inclusion in the regression analysis to reduce skewness and heteroscedasticity.

## 3 Results

### 3.1 Demographic and Clinical Characteristics of Dengue Patients

All patients and healthy controls belonged to the Kinh ethnic group and were residents of Hanoi metropolitan area living in various communes. The patient group comprised 127 males and 111 females, with a median age of 47 years (range: [14–87] years). The healthy control group consisted of 66 male and 52 female with the median age of 45 years (range: [25–56] years). Detailed demographic and clinical data of the recruited patients are presented in Table 1. There were no significant differences in age and sex between patient severity groups, or between patients and controls (Table 1).

**Table 1:** Characteristics of dengue patients.

	Dengue without warning signs (*n* = 103)	Dengue with warning signs (*n* = 122)	Severe dengue (*n* = 13)	*p* value
**Demographic data**				
Age (years)	46 [14–87]	49 [17–83]	49 [21–82]	0.627
Gender (male/female)	56/47	65/57	6/7	0.855
**Clinical manifestation**				
Day of disease (days)	3 [1–5]	5 [1–8]	5 [4–7]	< 0.001*
Headache	97 (94.2%)	106 (86.9%)	12 (92.3%)	0.192
Retro‐ocular pain	54 (52.4%)	84 (68.9%)	12 (92.3%)	0.003*
Myalgia	75 (72.8%)	92 (75.4%)	10 (76.9%)	0.637
Arthralgia	62 (60.2%)	85 (69.7%)	11 (84.6%)	0.116
Rash	13 (12.6%)	70 (57.4%)	6 (46.2%)	< 0.001*
Abdominal pain	0 (0%)	20 (16.4%)	5 (38.5%)	< 0.001*
Vomit	15 (14.6%)	31 (25.4%)	6 (46.2%)	0.008*
Lethargy	0 (0%)	1 (0.8%)	4 (30.8%)	< 0.001*
Edema	0 (0%)	26 (21.3%)	6 (46.2%)	< 0.001*
Hepatomegaly	0 (0%)	3 (2.5%)	0 (0%)	0.248
Shock	0 (0%)	0 (0%)	5 (38.5%)	< 0.001*
Respiratory distress	0 (0%)	0 (0%)	6 (46.2%)	< 0.001*
**Bleeding manifestations**	15 (14.9%)	102 (83.6%)	10 (76.9%)	< 0.001*
Subcutaneous	15 (14.9%)	82 (67.2%)	8 (61.5%)	< 0.001*
Mucosal	0 (0%)	64 (52.5%)	6 (46.2%)	< 0.001*
Severe	0 (0%)	0 (0%)	4 (30.8%)	< 0.001*
**Laboratory tests**				
Erythrocyte ×10^6^/μL	4.83 [3.66–6.26]	5.14 [3.97–7.62]	5.08 [2.76–5.89]	< 0.001*
Haemoglobin g/L	146 [110–187]	153 [113–190]	150 [70.0–172]	0.003*
Haematocrit	43 [31.8–53.4]	44.7 [34.8–60.5]	43.7 [21.4–51.6]	0.001*
Platelet ×10^3^/μL	138 [14.0–384]	21.0 [4.00–228]	29.0 [4.00–125]	< 0.001*
Leucocyte ×10^6^/μL	4.08 [1.27–16.9]	3.71 [0.96–11.6]	4.70 [1.45–10.5]	0.762
Neutrophile ×10^6^/μL	2.56 [0.47–13.1]	1.77 [0.45–6.21]	1.97 [0.81–7.70]	< 0.001*
Lymphocyte ×10^6^/μL	0.69 [0.17–3.56]	0.99 [0.22–4.09]	1.03 [0.31–2.81]	0.003*
Monocyte ×10^6^/μL	0.39 [0.06–1.35]	0.31 [0.05–2.53]	0.39 [0.10–0.74]	0.189
Eosinophile ×10^6^/μL	0.01 [0–0.21]	0.02 [0–0.45]	0.01 [0–0.07]	0.036*
Basophile ×10^6^/μL	0.03 [0–1.18]	0.07 [0–1.76]	0.09 [0.01–0.26]	< 0.001*
LUC ×10^6^/μL	0.10 [0.02–3.49]	0.36 [0.02–5.82]	0.40 [0.05–2.45]	< 0.001*
Neutrophile %	65.8 [18.7–93.4]	49.7 [17.3–82.3]	56.5 [26.1–76.1]	< 0.001*
Lymphocyte %	19.2 [2.5–72.8]	27.9 [6.1–56.3]	21.1 [6.8–53.0]	< 0.001*
Monocyte %	9.10 [1.6–20.9]	7.80 [2.9–36.6]	8.50 [3.6–11.8]	0.098
Eosinophile %	0.40 [0–4.00]	0.65 [0–11.6]	0.20 [0.10–2.30]	0.002*
Basophile %	0.60 [0–11.3]	2.20 [0–15.1]	1.40 [0.60–4.70]	< 0.001*
LUC %	2.70 [0.4–35.0]	10.0 [1.30–55.6]	6.55 [3.30–26.1]	< 0.001*
AST U/L	46.3 [17.3–350]	111 [16.0–1040]	186 [31–11100]	< 0.001*
ALT U/L	35.3 [8.00–503]	66.2 [8.20–636]	90 [25.6–2190]	< 0.001*

*Note:* Variables were summarized in percentage (%) or median with [range]. *p* values were calculated by Chi‐square and Kruskal‐Wallis test.

Abbreviations: ALT, alanine aminotransferase; AST, aspartate aminotransferase; LUC, large unstained cells.

* Statistically significant.

All patients were confirmed dengue‐positive by RT‐PCR. Patients with DWS and SD were hospitalized later in the disease course (median 5 days) compared to those with DF (median 3 days). Patients with DWS and SD more frequently presented with rash and bleeding manifestations compared to those with DF (Table 1).

### 3.2 Laboratory Parameters of Dengue Patients

Patients' laboratory parameters are summarized in Table 1. There was no difference in the total leukocyte counts between the three severity grades. DF patients had lower lymphocyte counts (median [range]: 0.69 [0.17–3.56] × 10^6^/μL) compared to DWS (median [range]: 0.99 [0.22–4.09] × 10^6^/μL) (*p* = 0.001) (Table 1 and Supporting Information S1: Table S1). In contrast, neutrophil and platelet counts were significantly higher in DF (NEU median [range]: 2.56 [0.47–13.1] × 10^6^/μL; PLT median [range]: 138 [14.0–384] × 10^3^/μL) compared to DWS (NEU median [range]: 1.77 [0.45–6.21] × 10^6^/μL; PLT median [range]: 21.0 [4.00–228] × 10^3^/μL) (Table 1 and Supporting Information S1: Table S1). In addition, liver enzymes including AST and ALT were higher in DWS (AST median [range]: 111 [16.0–1040] U/L; ALT median [range]: 66.2 [8.20–636] U/L) compared to DF (AST median [range]: 46.3 [17.3–350] U/L; ALT median [range]: 35.3 [8.00–503] U/L) (Table 1 and Supporting Information S1: Table S1).

### 3.3 sHLA‐G Plasma Levels in Healthy Controls and in Dengue Patients

**Figure 1:**
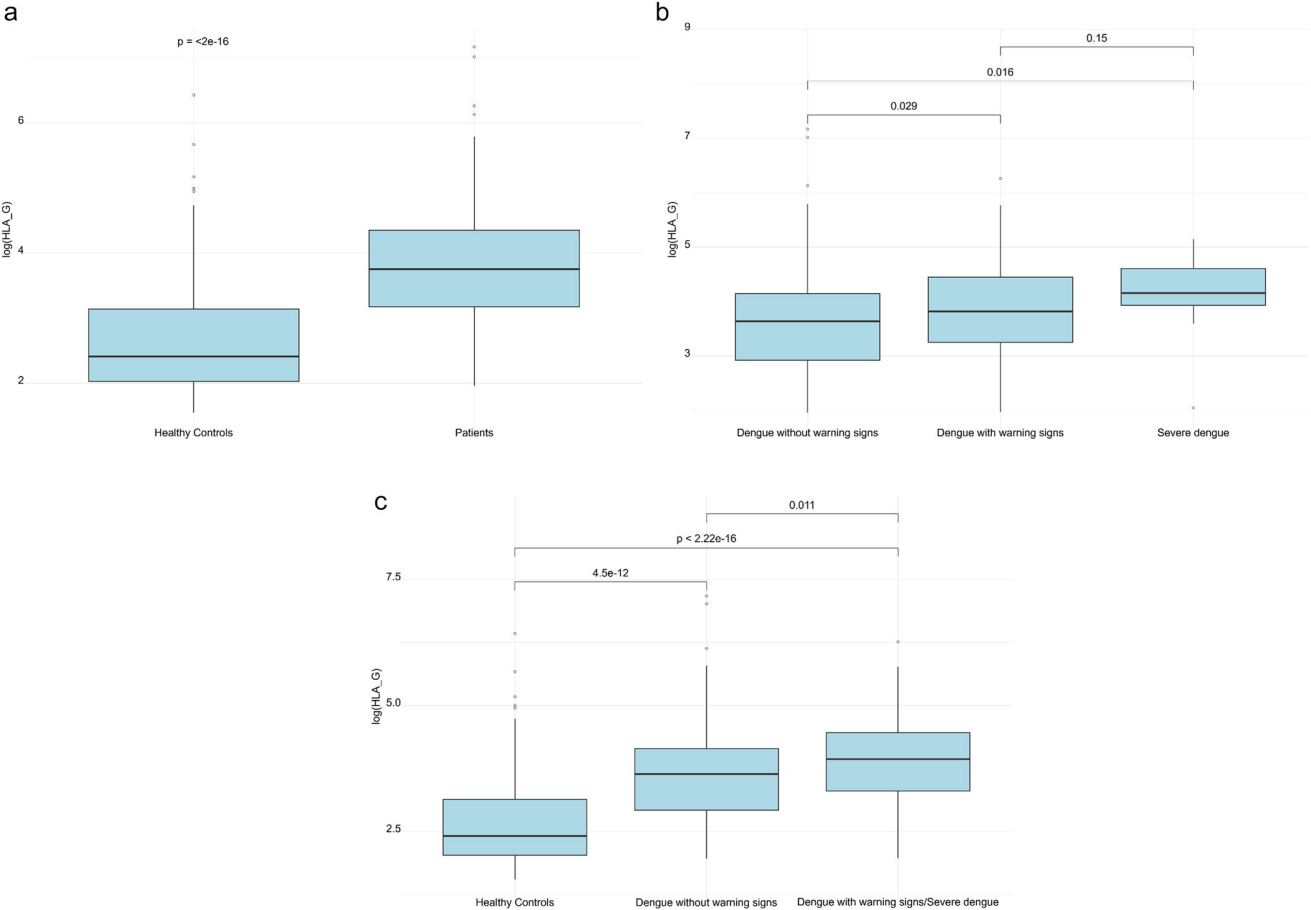
sHLA‐G plasma levels in dengue patients and its clinical relevance. (a) sHLA‐G plasma levels in dengue patients and healthy controls. (b) sHLA‐G plasma levels in dengue patients of varying severity. (c) sHLA‐G plasma levels in dengue patients by medical monitoring requirements. sHLA‐G plasma levels were determined using a commercially available ELISA as described in Methods. The data are provided as box plots as log‐transformed sHLA‐G (U/mL).

**Figure 2:**
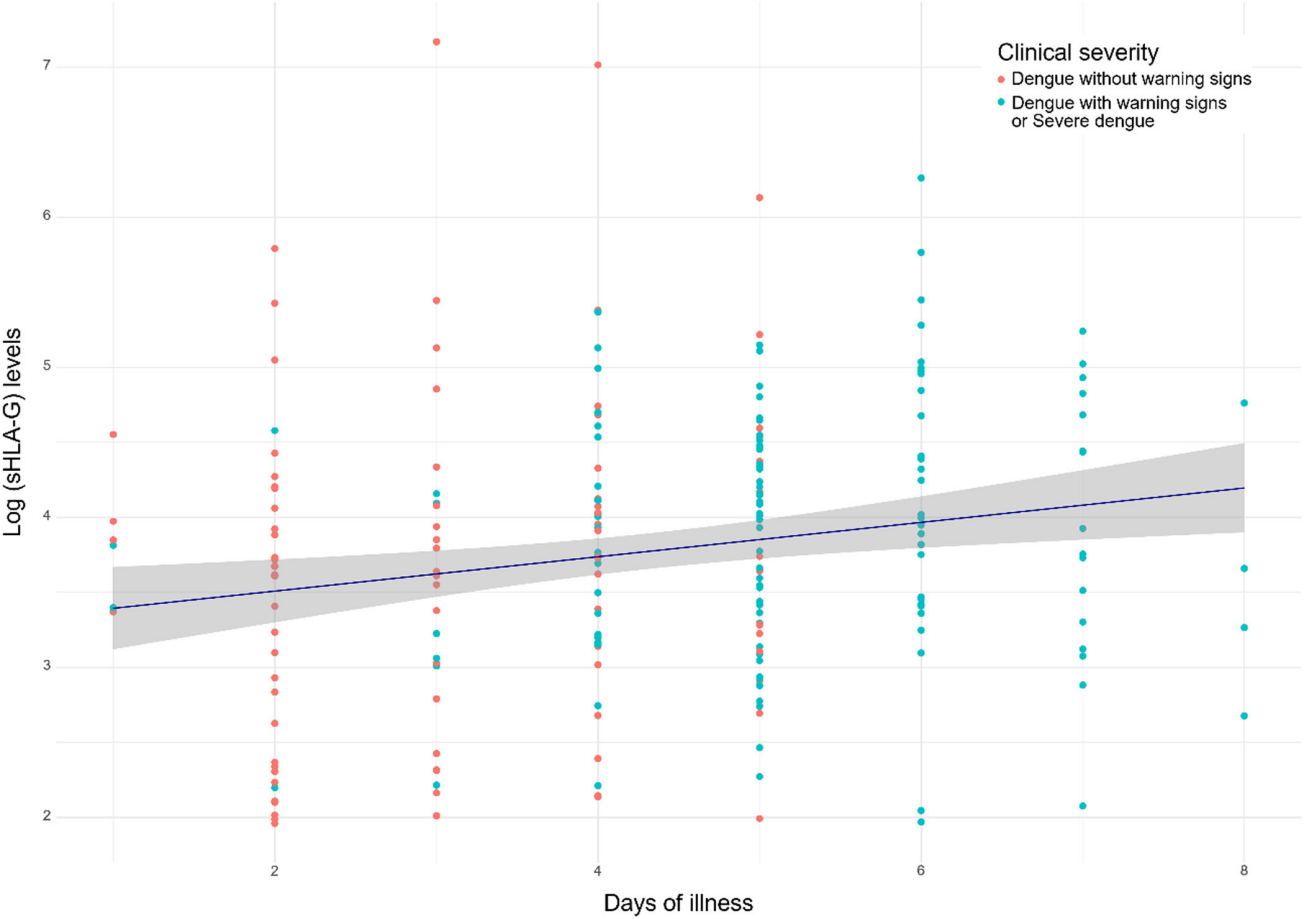
sHLA‐G levels and day of illness multiple linear regression was performed adjusting disease severity to estimate the association between log‐transformed sHLA‐G levels and days of illness.

The levels of sHLA‐G were significantly higher in dengue patients (median [range]: 42.7 [7.10–1300] U/mL) compared to healthy controls (median [range]: 11.1 [4.7–620] U/mL) (*p* < 0.001) (Figure 1a). In addition, sHLA‐G levels differed significantly across severity groups: between DWS (median [range]: 45.6 [7.17–525] U/mL) and DF (median [range]: 38.0 [7.10–1300] U/mL) (*p* = 0.029); and between SD (median [range]: 63.9 [7.73–172] U/mL) and DF (*p* = 0.016) (Figure 1b and Table 2). Notably, significantly higher sHLA‐G levels were observed in patients requiring close medical monitoring (DWS/SD) (median [range]: 51.0 [7.17–525] U/mL) compared to those with dengue fever (DF) (median [range]: 38.0 [7.10–1300] U/mL), (*p* = 0.011). Further stratification into DWS and SD subgroups revealed no significant difference in sHLA‐G levels. Multiple linear regression adjusting for age, sex, severity was performed to estimate the association between sHLA‐G levels (log‐transformed) and days of illness. The results showed a positive association between two variables (*β* = 0.1, *p* = 0.033) (Supporting Information S1: Table S2 and Figure 2).

**Table 2:** Levels of sHLA‐G in dengue patients of varying clinical severities.

	Dengue without warning signs (*n* = 103)	Dengue with warning signs (*n* = 122)	Severe dengue (*n* = 13)	*p* value
Mean (SD)	77.8 (174)	68.1 (68.2)	76.2 (42.4)	0.013*
Median [Min, Max]	38.0 [7.10–1300]	45.6 [7.17–525]	63.9 [7.73–172]	

	Dengue without warning signs (*n* = 103)	Dengue with warning signs and severe dengue (*n* = 135)	Healthy controls (*n* = 118)	
Mean (SD)	77.8 (174)	68.9 (66.1)	29.1 (67.1)	< 0.001*
Median [Min, Max]	38.0 [7.10–1300]	51.0 [7.17–525]	11.1 [4.70–620]	

*Note: p* values were calculated by Kruskal‐Wallis test.

* Statistically significant.

### 3.4 Plasma Levels of sHLA‐G Correlated With Laboratory Parameters in Dengue

Thirteen laboratory parameters: WBC, NEU, LYM, MONO, EOS, BASO, LUC, RBC, Hb, HCT, PLT, AST and ALT were included in the analysis (Table 3). While LUC and liver enzymes positively correlated with sHLA‐G levels in all dengue patients (LUC: *r* = 0.21, *p* = 0.004; AST: *r* = 0.21, *p* = 0.001; ALT: *r* = 0.14, *p* = 0.038), PLT and NEU showed inverse correlations (PLT: *r* = −0.21, *p* = 0.001; NEU: *r* = −0.16, *p* = 0.013) (Table 3). In addition, LYM and LUC inversely correlated with sHLA‐G levels in severe cases (LYM: *r* = −0.78, *p* = 0.002; LUC: *r* = −0.81, *p* = 0.005) (Table 3).

**Table 3:** Correlation of sHLA‐G levels with laboratory parameters.

	WBC	NEU	LYM	MONO	EOS	BASO	LUC	RBC	Hb	HCT	PLT	AST	ALT
**All patients**													
*R*	−0.04	−**0.16**	0.11	−0.05	0.03	0.13	**0.21**	0	0.04	0.09	−**0.21**	**0.21**	**0.14**
*p* value	0.557	0.013*	0.097	0.488	0.7	0.051	0.004*	0.975	0.518	0.184	0.001*	0.001*	0.038*
**DF**													
*R*	−**0.2**	−**0.21**	0.03	−0.13	−**0.23**	−0.03	0.09	−0.08	−0.09	−0.01	−0.1	0.06	0.01
*p* value	0.041*	0.032*	0.731	0.199	0.019*	0.763	0.444	0.418	0.341	0.917	0.301	0.547	0.883
**DWS**													
*R*	0.14	−0.04	0.18	0.11	**0.26**	**0.2**	0.2	0	0.12	0.12	−0.14	0.13	0.05
*p* value	0.132	0.645	0.06	0.259	0.006*	0.032*	0.049	0.992	0.225	0.195	0.112	0.166	0.575
SD													
*R*	−0.44	−0.23	−**0.78**	−0.28	−0.01	−0.39	−**0.81**	0	0.03	−0.02	−0.2	0.07	0.19
*p* value	0.135	0.448	0.002*	0.352	0.976	0.188	0.005*	1	0.922	0.943	0.502	0.817	0.529

*Note:* Significant correlations are highlighted in bold.

Abbreviations: ALT, alanine aminotransferase; AST, aspartate aminotransferase; BASO, basophile; DF, dengue without warning signs; DWS, dengue with warning signs; EOS, eosinophile; Hb, haemoglobin; HCT, haematocrit; LUC, large unstained cells; LYM, lymphocyte; MONO, monocyte; NEU, neutrophile; PLT, platelet; R, Spearman correlation coefficient; RBC, erythrocyte; SD, severe dengue; WBC, Leucocyte.

* Statistically significant.

## 4 Discussion

The severity of dengue primarily depends on immunopathogenic mechanisms, where an impaired immune response not only contributes to the progression of the disease, but also hinders the elimination of DENV and disease complications [5]. HLA‐G is known for its tolerogenic role suppressing the activity of effector cells, in particular NK cells and CD8+ cytotoxic T lymphocytes [12] and thus might be potentially involved in the pathogenesis of dengue. Based on this assumption, our study aimed to investigate the association between sHLA‐G and dengue in Vietnamese patients. The results suggested that sHLA‐G levels were associated with dengue and might serve as a marker for disease severity.

In our study, plasma levels of sHLA‐G were significantly higher in dengue‐confirmed patients, compared to healthy controls. The expression of HLA‐G is restricted to certain tissues under physiological conditions, but is upregulated during pregnancy and in pathological conditions such as tumours, viral infections and inflammatory diseases [13]. Soluble HLA‐G proteins are formed by two mechanisms: alternative splicing and proteolytic release mediated by metalloproteinases. Among two mechanisms, it is likely that proteolytic release is a backup to alternative splicing to control specific immunomodulatory functions [14]. The production of sHLA‐G is regulated by various factors, including genetic, hormonal, pathological and immunological signals. For example, Park and co‐authors reported that proteolytic release is particularly pronounced in pathological conditions, in which mutations occur at the splice sites of the HLA‐G gene [14].

Previous studies have suggested that the expression of sHLA‐G during dengue pathogenesis can be modulated by various mechanisms. In particular, interleukin‐10, interferon‐gamma and matrix metalloproteinases (MMP‐2 and MMP‐9), all of which are elevated in severe dengue, have been shown to enhance the production of sHLA‐G [15, 16, 17]. Notably, MMP‐2 and MMP‐9 levels are significantly elevated in severe dengue and have been associated with impaired immune responses and increased disease complications in affected patients [18]. These findings suggest that sHLA‐G may contribute to immune suppression during dengue through multiple mechanisms, potentially leading to reduced activity of NK cells, CD8⁺ T cells, and regulatory T cells.

In contrast, Renata and co‐authors observed decreased sHLA‐G levels during the acute phase arbovirus infection in Brazilian patients compared to the recovery phase that reflects the stable state of the patient's immune system [6]. Nevertheless, patients in this study were confirmed to have Zika and chikungunya with neurological complications. These differences in viral aetiology and clinical presentation may account for the discrepancy in sHLA‐G profiles compared to our dengue‐infected cohort. However, our findings align with other studies reporting elevated sHLA‐G levels in patients infected with human cytomegalovirus, herpes, hepatitis and SARS‐CoV‐2 viruses compared to healthy controls [19]. In addition, polymorphisms within the HLA‐G gene can influence plasma concentrations, potentially varying across different populations [20, 21].

Time of symptom onset is recognized as a key determinant of clinical severity in dengue [22]. In our study, multiple linear regression analysis revealed a positive association between the number of illness days and plasma sHLA‐G levels after adjusting for age, sex and disease severity. These findings suggest that sHLA‐G levels increase during the course of dengue infection, underscoring the dynamic nature of this biomarker. This also highlights the importance of considering illness duration when evaluating sHLA‐G as a potential indicator of disease progression. Despite our findings demonstrate a clear association between sHLA‐G levels and clinical severity indicators, the data should be interpreted as correlative. Further longitudinal studies are needed to determine the temporal dynamics of sHLA‐G elevation and its potential mechanistic role in dengue pathogenesis.

In addition, our study revealed correlations between sHLA‐G levels and key parameters for dengue, such as NEU, PLT, AST and ALT [3, 22]. These findings highlight the potential added value of integrating sHLA‐G with routine clinical markers to support triage and enhance understanding of the disease course. Lymphocytes are critical for antiviral immunity and their depletion, a hallmark of severe dengue and other viral infections, is associated with impaired immune response and increased infection consequences [23]. In particular, a strong inverse correlation between sHLA‐G levels and total lymphocyte counts was observed in severe cases. These data suggested that elevated sHLA‐G may contribute to lymphocyte depletion by exerting immune suppressive effects, such as inducing apoptosis or inhibiting lymphocyte proliferation, thereby exacerbating immune dysfunction and contributing to disease severity [24].

Also, large unstained cell (LUC) count was inversely correlated with sHLA‐G level in severe patients. LUC includes large, activated lymphocytes and other atypical cells, such as virocytes, blasts cells and hematopoietic stem cells. LUC count is considered a potential indicator of a patient's immune response status during viral infections [25, 26]. A reduced LUC count may indicate impaired immune activation and is thus associated with increased infection severity. Correlations between LUC and CD8+ as well as CD4 + T cells were also noted in HIV patients [27] and LUC was suggested to be a predictive biomarker for hematologic toxicities in cancer and hematologic malignancies [28]. The observed negative correlation between LUCs and sHLA‐G levels in patients with severe clinical manifestations may reflect a reduction in activated lymphocytes in response to elevated sHLA‐G suggesting a potential role for sHLA‐G in suppressing immune defense. Nonetheless, a significant positive correlation between LUC count and sHLA‐G levels was found when all patients with varying degrees of severity were analysed together suggesting that LUC counts may respond differently to changes in sHLA‐G levels across different stages and severities.

Notably, strong and statistically significant correlations between sHLA‐G levels and laboratory parameters, including lymphocyte and large unstained cell counts, were observed exclusively in severe dengue cases. However, these findings must be interpreted with caution due to the small number of patients in the severe dengue group (*n* = 13). This represents a key limitation of our study, as it limits the statistical power and generalizability of the subgroup analyses. Despite this constraint, our results provide preliminary evidence that molecular immune checkpoints, such as sHLA‐G, may contribute to the complex immunopathogenesis of severe dengue and warrant further investigation in larger cohorts. Furthermore, the possibility to assess the level of sHLA‐G only at one time point in each patient limited the understanding of sHLA‐G dynamics.

## 5 Conclusion

Immune inhibitors potentially have an important role in the pathogenesis of dengue. In conclusion, our findings suggest that sHLA‐G levels are associated with dengue severity, highlighting its potential as a marker for future clinical monitoring and early identification of severe cases.

## Author Contributions

Barbara Seliger and Thirumalaisamy P. Velavan designed, supervised the study and contributed to the study materials and assays. Le Huu Song and Thirumalaisamy P. Velavan were involved in the conceptualization of the study. Do Duc Anh and Anja Mueller performed the experimental procedures, the statistical analysis and validation of the results. Nguyen Trong The and Le Huu Song recruited the patients and contributed to the investigation materials for sampling procedures. Do Duc Anh wrote the first draft. Do Duc Anh, Thirumalaisamy P. Velavan and Barbara Seliger reviewed the first draft. All authors have read and approved the manuscript.

## Ethics Statement

All study participants provided signed informed consent before enrolment. The Institutional Review Board of the 108 Military Hospital and the University of Tübingen approved the study, titled “Host and Viral Factors Influencing Dengue Severity and Susceptibility” (Ethics Approval No. 274/2022B02). The study complies with the Nagoya Protocol, and authorization for the use of genetic resources in Germany was obtained from the Vietnamese Ministry of Natural Resources and Environment (Reference No. 2995/QĐ‐BTNMT). All procedures followed GCP/GCLP guidelines.

## Conflicts of Interest

The authors declare no conflicts of interest.

## Supporting information


**Supplementary 1:** *p* value from post hoc Dunn test. **Supplementary 2:** Multiple linear regression for sHLA‐G level and day of illness.

## Data Availability

Data sharing not applicable to this article as no datasets were generated or analysed during the current study.
