# Supplementary material for: Elevated Soluble HLA‐G Levels Associate With Dengue Severity in Vietnamese Patients
Source: J Med Virol. 2025 Sep 5;97(9):e70594. doi: 10.1002/jmv.70594 (PMC12412082; doi:10.1002/jmv.70594)
Supplement: Supplementary file 1 — Supplementary 1: p value from post hoc Dunn test. Supplementary 2: Multiple linear regression for sHLA‐G level and day of illness. [file JMV-97-e70594-s001.docx]

**Supplementary 1. p value from post hoc Dunn test**

|  | **WBC** | **NEU** | **LYM** | **MONO** | **EOS** | **BASO** | **LUC** | **RBC** | **Hb** | **HCT** | **PLT** | **AST** | **ALT** |
| --- | --- | --- | --- | --- | --- | --- | --- | --- | --- | --- | --- | --- | --- |
| DF vs DWS | NA | <0.001* | 0.001* | NA | 0.02* | <0.001* | <0.001* | <0.001* | 0.001* | <0.001* | <0.001* | <0.001* | <0.001* |
| DF vs SD | NA | 0.727 | 0.258 | NA | 1 | 0.028* | 0.023* | 0.776 | 0.982 | 1 | <0.001* | <0.001* | <0.001* |
| DWS vs SD | NA | 0.397 | 1 | NA | 0.322 | 1 | 1 | 0.354 | 0.393 | 0.234 | 1 | 0.497 | 0.373 |

^WBC: Leucocyte; NEU: Neutrophile; LYM: Lymphocyte; MONO: Monocyte; EOS: Eosinophile; BASO: Basophile; LUC: Large Unstained Cells; RBC: Erythrocyte; Hb: Haemoglobin; HCT: Haematocrit; PLT: Platelet; AST: Aspartate Aminotransferase; ALT: Alanine Aminotransferase. DF: dengue without warning signs; DWS: dengue with warning signs; SD: Severe dengue. NA: not applicable. *: statistically significant.^

**Supplementary 2. Multiple linear regression for sHLA-G level and day of illness**

|  | **Estimate** | **Standard error** | **t value** | **p value** |
| --- | --- | --- | --- | --- |
| Intercept | 3.392 | 0.247 | 13.749 | <0.001* |
| Days of illness | 0.104 | 0.049 | 2.137 | 0.033* |
| Age | -0.001 | 0.004 | -0.186 | 0.853 |
| Sex (Male) | -0.132 | 0.122 | -1.086 | 0.279 |
| Dengue with warning signs | 0.042 | 0.157 | 0.266 | 0.790 |
| Severe dengue | 0.299 | 0.292 | 1.026 | 0.306 |

^Estimate, Standard Error, t-value and p values were calculated by multiple linear regression. *: statistically significant.^
